# Supplementary material for: Cloud BioLinux: pre-configured and on-demand bioinformatics computing for the genomics community
Source: BMC Bioinformatics. 2012 Mar 19;13:42. doi: 10.1186/1471-2105-13-42 (PMC3372431; doi:10.1186/1471-2105-13-42)
Supplement: Additional file 1 — Supplementary 1 Cloud BioLinux software documentation in the form of a mini, self-contained website. Users need to download and uncompress the .zip file, and open through a web browser the "index.html" file available on the main directory. (ZIP 1823 kb). [file 1471-2105-13-42-S1.ZIP › Cloud-BioLinux-Package-Documentation/docs/gromacs.html]

Bio-Linux Software Documentation Pages

Back to search form

## gromacs

|  |  |
| --- | --- |
| Name | gromacs |
| Description | gromacs is a versatile package to perform molecular dynamics. It is designed to simulate the Newtonian equations of motion for systems with hundreds to millions of particles.  It is primarily designed for biochemical molecules like proteins and lipids that have a lot of complicated bonded interactions.  The Gromacs package is prepared by Debian-Med and can be installed in Bio-Linux by typing: `sudo apt-get install gromacs` For further information about using Gromacs please see the remote documentation links |
| Homepage | http://www.gromacs.org/ |
| Remote Documentation | http://www.gromacs.org/   http://manual.gromacs.org/ |
